# Orthognathic Surgery as Class III Skeletal Treatment in a 31-Year-Old Female with Mandible Prognathism: A Case Report

**DOI:** 10.1055/s-0043-1761453

**Published:** 2023-03-28

**Authors:** Seto Adiantoro Sadputranto, Arlette Suzy Setiawan, Ani Melani Maskoen, Avi Laviana, Endang Sjamsudin

**Affiliations:** 1 Doctoral Candidate Study Program Faculty of Dentistry, Universitas Padjadjaran, Bandung, Indonesia; 2 Department of Pediatric Dentistry, Faculty of Dentistry, Universitas Padjadjaran, Bandung, Indonesia; 3 Department of Oral Biology Faculty of Dentistry, Universitas Padjadjaran, Bandung, Indonesia; 4 Department of Orthodontic Faculty of Dentistry, Universitas Padjadjaran, Bandung, Indonesia; 5 Department of Oral Maxillofacial Surgeon Faculty of Dentistry, Universitas Padjadjaran, Bandung, Indonesia

**Keywords:** mandible prognathism, orthognathic surgery, malocclusion skeletal class III

## Abstract

Mandible prognathism or malocclusion skeletal class III is facial deformities. These deformities can affect orofacial function, such as mastication, speech, and function of the temporomandibular joint. Besides the physical effects of these deformities, the psychosocial impact on the individual is often essential, and such deformities can affect the quality of life and self-confidence. Orthognathic surgery is designed to correct these deformities because these deformities could not have been corrected by only orthodontic treatment. Therefore, at Hasan Sadikin General Hospital, orthognathic surgery is the treatment choice for mandibular prognathism or malocclusion skeletal class III. In this case report, we present a 31-year-old female with mandibular prognathism, difficulty in closing her mouth and anterior open bite. Surgery was performed by Le Fort 1 osteotomy for advancing maxilla and bilateral sagittal split osteotomy for setback mandible. Two weeks after surgery, patient came back to the orthodontic department for occlusion treatment.

## Introduction

Orthognathic surgery aims are a treatment for class III skeletal or mandible prognathism. The purpose of this surgery for correction position maxilla and mandible with the goal of therapy is making betters functional and aesthetic. Orthognathic surgery involves an orthodontist for preoperative preparation and postoperative occlusion treatment. Surgery is performed to reposition the jaw, resulting in a more harmonious facial frame. Many studies show that orthognathic surgery has improved facial and dental aesthetics and improved function after treatment. Orthognathic surgery restores the occlusal function and the aesthetic by improving facial harmony. 1 2 3 Several research reported impact of orthognathic surgery including quality of life, psychologic, social live, physic, aesthetic and also function, before and after surgery. Based on World Health Organization, quality of life is individual perception about their position life in cultural context and value system connection to their destination of life, hope, standard of life, and worries. 1 2 3 4

Before surgery, plan and treatment result should be discussed between surgeon and orthodontist. The orthodontist and surgeon must make a deal about diagnosis and surgery and orthodontic plan. The surgery plan is important to make a stable jaws and harmonious profile. Orthodontic treatment before and after surgery is to correct dental skeletal deformity with orthognathic surgery. Conventional plan of surgery involves facial analysis, cephalometry, model casting to make occlusion when doing surgery with intermediate and final wafer. In the recent years, use of technology computer for surgery planning has increased. Surgical goal becomes easy computer is used for surgery planning. 5 6

There are three types of osteotomies used for orthognathic surgery: first, bilateral sagittal split osteotomy (BSSO) with an incision in the intraoral region of the mandible to gain access to the posterior region of left and right mandibles; second, vertical ramus osteotomy (VRO); the incision is made in the posterior area to gain access to the mandibular ramus area. The vertical ramus cutting is done with a saw. Third, for the maxilla with Le Fort 1 osteotomy technique, an intraoral incision is made in the vestibule left first molar to the right first molar. Le Fort 1 maxillary bone is cut using a saw. 4

The relationship between the lower and upper lips often lacks contact or no contact occlusion in mandibular prognathism due to crossbite anterior teeth bites or open bites in the anterior or lateral occlusal area. Side effects are impaired speech, chewing, and aesthetic functions. 5 Population Asian people have the highest prevalence of class III malocclusion. Malaysia and Chinese people have the highest prevalence, approximately 15.69 and 16.59%, respectively. Prevalence for Indian people is lower than for other races; in the United States, it is 5% from orthodontic patients and only 1% from all of the population. Environmental and genetic factors have contributed to etiologic class III malocclusion; thus, class III malocclusion is very broad and complex. Therefore, it is imperative to determine the class III dental or skeletal malocclusion so that appropriate treatment can be performed. 6 This article aims to show that orthognathic surgery provides maximum results for treating skeletal class III cases with mandibular prognathism.

## Case Report

**Fig. 1 (A):**
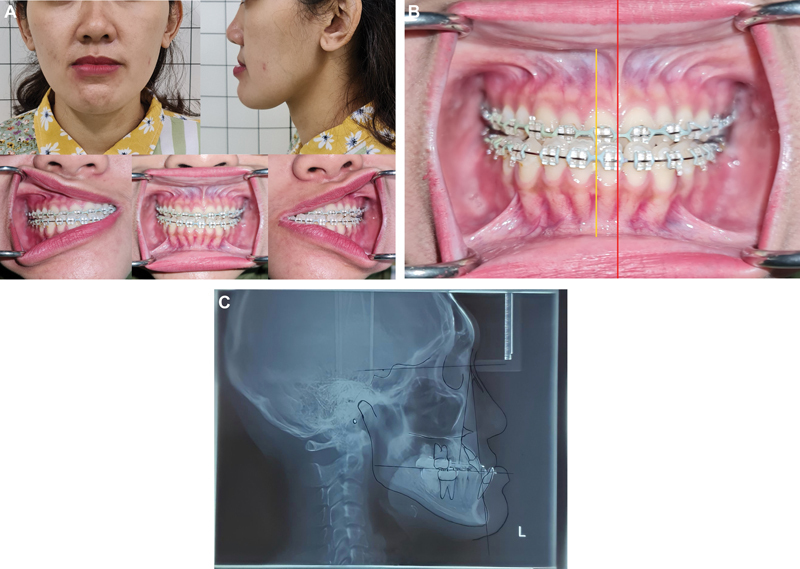
Preoperative extraoral and intraoral status. **(B)** The right mandible midline deviated. ( **C** ) Cephalogram.

A 31-year-old female came to the oral and maxillofacial department at Hasan Sadikin General Hospital. The patient's chief complaint was difficulty with speech and chewing; her profile was concave, and the mandible was protruded ( Fig. 1A ).

Clinical findings of the patient showed that the facial profile was disproportional of the lower jaw, class III facial profile with a concave profile. Occlusion was malocclusion class III, anterior crossbite, and overjet was −6 mm, with deviated midline mandible to the correct 8 mm. Still, without canting of the maxilla ( Fig. 1B ), periodontal problems were not found; however, there was a history of temporomandibular joint (TMJ) problem with uncomfortable sense during mastication and mandibular shifting after yawning. SNA, SNB, and ANB values from analysis of the cephalogram ( Table 1 ) showed mandible prognathism. Therefore, based on clinical findings and cephalogram analysis, the diagnosis is malocclusion class III skeletal with mandibular prognathism and planned for orthognathic surgery to obtain a maximum functional and aesthetic result.

**Table 1:** Cephalometric analysis ( Fig. 1C )

Skeletal analysis			
Anteroposterior	Initial	Final	Normal
SNA	81 degrees		82 ± 2 degrees
SNB	93 degrees		80 ± 2 degrees
ANB	–12 degrees		2 ± 2 degrees
FMPA	42 degrees		25–30 degrees

Abbreviations: SNA, angle form by the intersection of sella-nasion and nasion-A lines; SNB, angle formed by the intersection of sella-nasion and nasion-B lines; ANB, angle formed by intersection nasion-A and nasion-B lines; FMPA, Frankfort-mandibular plane angle.

**Fig. 2:**
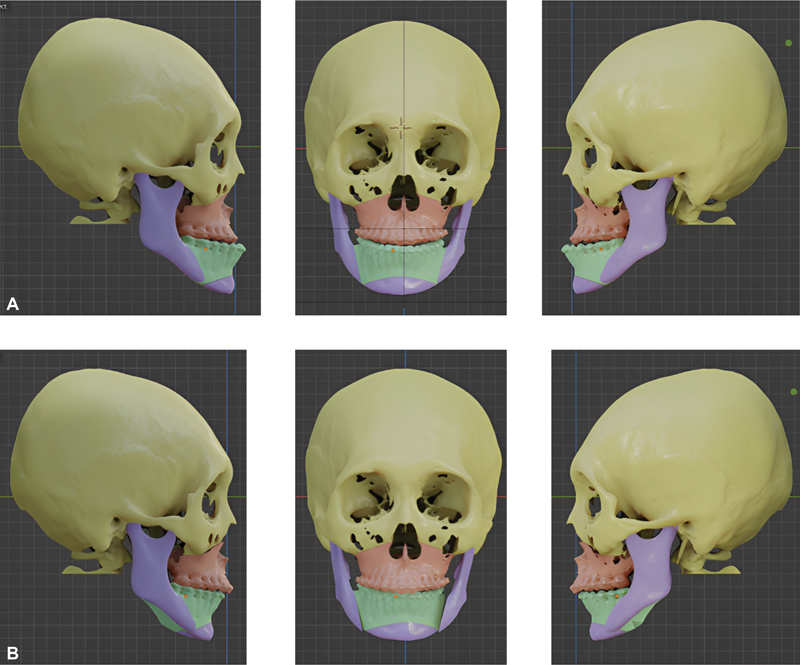
Three-dimensional planning program. **(A)** Design planning maxilla advancement 3 mm. **(B)** Design planning mandible backward 5 mm.

**Fig. 3:**
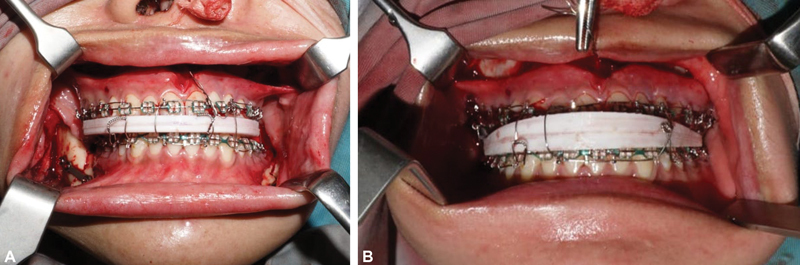
**(A)** Intermediate wafer—used to find position maxilla forward 3 mm; **(B)** final wafer—used to find the position mandible backward 5 mm.

Based on the clinical findings, it was decided to execute an osteotomy Le Fort 1 and BSSO with maxillary advancement up to 3 mm and mandibular setback up to 5 mm. Design planning of surgery besides tracing on the cephalogram, using a 3D planning program, so that it can be concluded that the maxilla is 3mm forward ( Fig. 2A ) and the lo6wer jaw is 5mmbackward ( Fig. 2B ). Wafers were used to obtain 3 mm positions for maxillary forward and 5 mm for mandibular backward in surgery. Wafers are used during the operation, and there are two wafers, an intermediate wafer and a final wafer. Intermediate wafers were used to get the maxillary position 3 mm forward ( Fig. 3A ); after placing the plate on the maxilla, the mandible was moved back up to 5 mm, using the wafer, the mandible was positioned back 5 mm ( Fig. 3B ).

**Fig. 4:**
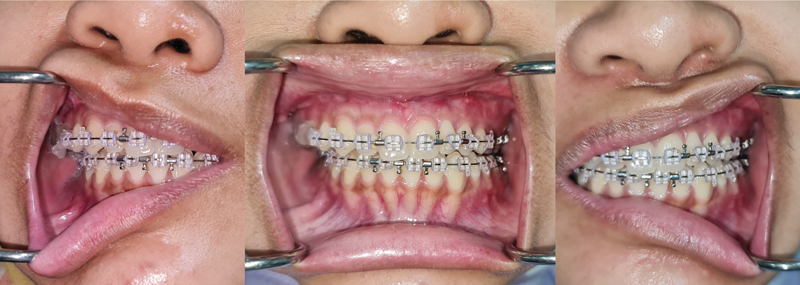
One month after surgery.

The procedure went well, and the patient was discharged 2 days later. The patient displayed a favorable facial profile and repair of the anterior open bite and overjet at the follow-up appointment 1 week later. Intermaxillary fixation (IMF) was used for the first 2 weeks following surgery to guarantee postoperative stability. The patient began a semisoft diet for an additional 3 weeks after the IMF was removed, and the recovery was under control. On the 1-month follow-up, it showed progress ( Fig. 4 ).

## Discussion

Orthognathic surgery is a procedure to improve the relationship between the upper and lower jaws and the profile of the face. The orthognathic procedure involves an orthodontist before and after surgery—orthognathic surgery to treat skeletal malocclusion that cannot be treated with conventional orthodontic treatment. Several abnormalities can be treated with orthognathic surgery: (1) Class II and class III skeletal angle malocclusions with anterior open bite and facial asymmetry, (2) TMJ abnormalities, (3) obstructive sleep apnea syndrome, (4) post-cleft lip and palate, (5) hemifacial microsomia, and (6) deformity or malocclusion due to trauma. 7

Abnormalities that often occur in cases of class III malocclusion with a prognathic mandible are facial asymmetry. Facial asymmetry occurs due to deviation of the maxilla or mandible or maxilla and mandible. Prognathic mandibles can result in skeletal class III malocclusions. In addition, skeletal class III malocclusions can be caused by a retruded maxilla or a combination of a prognathic mandible and a retrusive maxilla. Such abnormalities can only be treated by orthognathic surgery to correct occlusion and aesthetic abnormalities. 8 9

Mandibular prognathism is a condition when position of the mandible is more advanced than the cranial base and is categorized as a class III angle. Mandibular prognathism is a craniofacial bone disorder caused by genetics and environment, although genetics is more responsible for mandibular prognathism. Mandibular prognathism, also known as the Habsburg jaw, is a genetic disorder resulting in an underdeveloped mandible or a hypoplastic maxilla. 5 10 11

Soft tissue disorder is not the main etiologic in most skeletal class III cases. Moreover, soft tissue could support the alignment of incisive teeth, lessening the impact of the skeletal disorder on the teeth's alignment. The anterior oral seal of the upper and lower lip achieved compensation for dentoalveolar in class III malocclusion. This compensation does not happen in patients with increased vertical skeletal proportion where lips incompetence is found. The anterior oral seal was done by tongue to lower the lip seal. 12

Clinical examination, lateral cephalometric, and panoramic radiography were performed on all subjects affected by mandibular prognathism. The diagnosis of mandibular prognathism required at least two criteria: straight or concave facial profile; overjet less than 0 mm or edge-to-edge bite; class III molar and canine relationship and the ANB angle less than or equal to 0 degrees. 11 Table 1 shows the mandibular prognathism patient's clinical picture and cephalometric analysis SNB value of 90 degrees shows mandibular prognathism, and ANB value of −5 shows mandible is more protruded than the maxilla, and overjet is 9 mm. Thus, we can conclude that the diagnosis of this patient is skeletal class III with mandibular prognathism.

Orthognathic surgery aims to balance the facial and cranial structures by treating maxillary discrepancy using osteotomy procedures in the masticatory system. Maxilla and mandible alignment is done during orthognathic surgery to establish proper dental posture and repair facial and maxillomandibular irregularities. The maxilla–mandibular relationship and teeth alignment are balanced due to orthognathic surgical treatment. Sagittal split ramus osteotomy and intraoral VRO are the most often utilized techniques in orthognathic surgery. Orthognathic surgery is frequently used to treat underlying conditions that affect chewing, facial pain, and aesthetics. 4 13

**Fig. 5:**
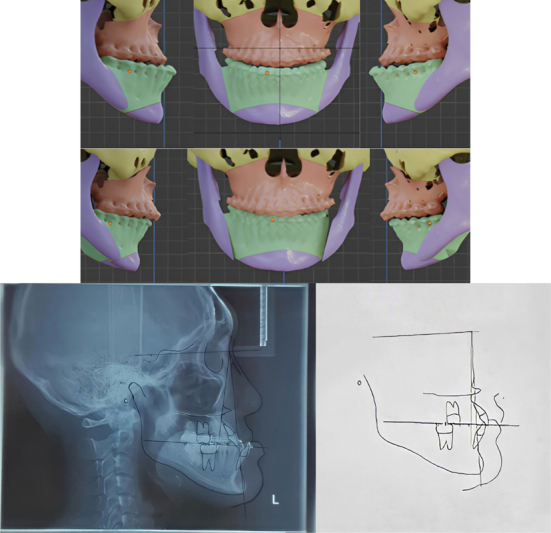
Cephalometry trace and three-dimensional planning program.

The patient, in this case, demonstrated significant class III skeletal and mandibula prognathism; of course, the impact of this case was on her appearance, mastication, and speech abnormality. Orthognathic surgery is required in skeletal class III cases with a prognathic mandible, as in this case, it cannot be solved by conventional orthodontic treatment. This case required orthognathic surgery because, based on cephalometric analysis and a three-dimensional planning program, the results were skeletal class III with a prognathic mandible. The surgery requires an action to bring forward the mandible by 4 mm and withdraw the mandible by 5 mm ( Fig. 5 ). Finding the occlusion during the surgery was difficult; thus, a wafer was necessary to find the occlusion when the maxilla was moved forward and the mandible was moved backward. Before surgery, correction of tooth alignment and levelling was performed, and the use of a wafer was a solution to solve this problem.

The main objective of orthognathic surgery is to repair maxilla protrusive condition and mandibular prognathism, malocclusion, temporomandibular disorders, and sleep apnea. Before the surgery, this patient did her orthodontic treatment in orthodontics department at Hasan Sadikin Hospital. Tooth 3.8 and 4.8 were extracted 6 months before the surgery because these teeth were in the position of the osteotomy line of BSSO. In addition, prior to the surgery, cephalometric tracing and wafer production were done.

The wafer is a device to transfer presurgical planning up until the post-surgical occlusion. Cephalometric planning is followed by the transmission of jaw movements to the actual physical manipulation of the dental models in the surgical wafer. When osteotomy segments are surgically moved and fixed, these acrylic interocclusal splints help physically align the upper and lower teeth. There are two interocclusal wafers: a final wafer for the desired postoperative occlusion and an intermediate wafer to guide maxillary osteotomy motions using the mandibular position as a baseline reference. The latter is commonly utilized for mandibular-only and bimaxillary osteotomies. 14

The Le Fort 1 osteotomy is frequently used to treat maxillomandibular abnormalities and malocclusions. Class III malocclusion is one of the most frequent reasons for undergoing a Le Fort 1 osteotomy. Le Fort 1 osteotomy with horizontal advancement helps the majority of patients to correct their malocclusion. 15 Starting with Le Fort 1 for the maxilla, after advancing the maxilla by 4 mm according to the planning before surgery, wafers and intramaxillary fixation were installed and ended with the L-shaped plates installed in the anterior and posterior maxilla.

The most frequent jaw surgery, either with or without upper jaw surgery, is the BSSO, a crucial part of orthognathic surgery. It is the most often used technique for mandibular advancement and can be used for a modest to the severe mandibular setback. BSSO is widely used for the mandible, starting by making an incision on the external oblique line through to the second molar mandibular region, then tissue split was done until the inferior mandibular cortex could be visualized, from ramus to coronoid process. Next, the Obwegeser refractor and Kocher clamp function to hold the soft tissue at the bottom of the mandibula, and then osteotomy was done from the top of the lingula to the anterior of the first or second molar region, continuing vertically to the bottom border of the mandible. After the split, the mandible could be retracted following the surgical plan using the final wafer to achieve ideal occlusion. The ideal occlusion could be fixed using a plate and screw after inter-maxillary wiring.

IMF was done 1-day post-surgery for 2 to 3 weeks, immobilizing the maxilla and mandibula and aiming for the bone union. IMF was released after 2 weeks, and orthodontic department consulted the patient. BSSO surgery complications include the inferior alveolar, retromandibular, or maxillary vein, the facial, mandibular, or maxillary artery, or the pterygoid venous plexus that may break as a result of tissue dissection, retraction, or separation during surgery. The structure of the maxillomandibular joint and the surgical approach may cause facial nerve damage. Hematomas were discovered in the gingival channel (0.09%), the submandibular area (0.18%), the submental area (0.18%), the cheek (0.27%), the floor of the mouth (0.58%), and the submandibular area. During the surgical procedure, several mechanical forces may cause condylar absorption. When using Le Fort I procedures, splitting and manipulating the maxilla may cause the nasal septum to deviate. Intraoperative bleeding, edema that persisted for a week or two after surgery, postoperative pain, nasal obstruction, and bilateral mandibular paresthesia were the postoperative problems. This case report has limitations in the absence of cephalometric analysis results after surgery due to pandemic restrictions.

## Conclusion

Orthognathic surgery aims to correct dentofacial deformity, including mandibular prognathism. Mandibular prognathism could be the effect of protruding mandible and retruded maxilla. This deformity could be corrected using orthognathic surgery by advancing the maxilla and setting back the mandible to restore mastication, speech, and esthetic function. Several problems presented in these patients with prognathic mandible were malocclusion, concave profile, and temporomandibular disorder that can be treated with orthognathic surgery.
